# Habitat Selection Patterns Suggest Competition Between Two Forest Rodent Species Along an Elevational Gradient

**DOI:** 10.1002/ece3.71490

**Published:** 2025-05-28

**Authors:** Ana Maria Benedek, Ioan Sîrbu

**Affiliations:** ^1^ Doctoral School in Ecology, Faculty of Biology University of Bucharest Bucharest Romania; ^2^ Biology and Ecology Research Center, Faculty of Sciences Lucian Blaga University of Sibiu Sibiu Romania

**Keywords:** bank vole, generalised linear mixed models (GLMM), habitat segregation, intra‐ and interspecific density, species coexistence, yellow‐necked mouse

## Abstract

Numerous mammal species exhibit dynamic habitat use and selection, choosing the more preferred habitats at low densities when these are more readily available. Habitat selection may also vary along elevation and in relation to other populations' densities, with interspecific competition potentially resulting in habitat segregation. However, little is known about these effects combined. We aimed to evaluate the differential habitat selection by the dominant rodents in relation to intra‐ and interspecific density along an elevational gradient. We used trapping data collected over 7 years (2000–2007) in forests of the Romanian Carpathian Mountains, where *Apodemus flavicollis* (yellow‐necked mouse) and *Clethrionomys glareolus* (bank vole) dominated small mammal communities. We hypothesised that (i) habitat segregation facilitates the coexistence of the two species, (ii) because of the differences in their thermic requirements, they have coexisted at high elevations for a shorter time, and therefore their competition is weaker here, resulting in less pronounced habitat segregation and (iii) the abundance of *C. glareolus* is negatively affected by the abundance of *A. flavicollis* , which is known to be more competitive. As predicted, the mouse and the vole showed significant opposite responses to most habitat characteristics, indicating habitat segregation as a mechanism of codominance in this relatively continuously forested landscape. As expected, the dissimilarity between their responses was smaller at high elevations, suggesting the recent elevational expansion of *A. flavicollis* . Contrary to our expectations, the mouse abundance was negatively affected by the vole abundance, but not vice versa. Our study provides insight into the interactions between *C. glareolus* and *A. flavicollis* in forests along the elevational gradient, suggesting asymmetrical competition and recent elevational expansion of the latter.

## Graphical abstract

We evaluate the differential habitat selection by rodents in relation to intra‐ and interspecific density along an elevational gradient using trapping data from the Romanian Carpathian Mountains. Our study provides insight into the interactions between *Apodemus flavicollis* (yellow‐necked mouse) and *Clethrionomys glareolus* (bank vole), the two dominant species in the researched small mammal communities, suggesting asymmetrical competition and recent elevational expansion of the former.

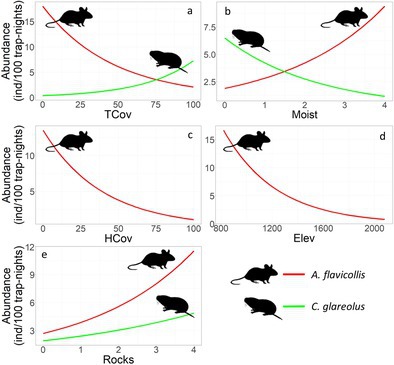

## 1 Introduction

How an animal uses the environment in which it lives and its resources has a paramount effect on all aspects of its life—feeding, physiology, survival and reproduction, among others, affecting ultimately its fitness and, therefore, having adaptive value (Kelt et al. 2019). Habitat use is the proportional description of the measurable habitat features a species uses. Generalist species use habitat types or environmental features proportionally to their availability (Villanueva‐Hernández et al. 2017). Habitat selection, the behaviour of disproportionate use of the available environmental features, implies perception and response to resources but also risks (predators, competitors). Strong habitat selection characterises habitat specialists (Rosenzweig 1981; Villanueva‐Hernández et al. 2017).

Habitat selection occurs at the individual level through animal behaviour. It is translated into the habitat niche, which results from adaptation—the effect of evolution through natural selection. Thus, it depends on physiological tolerance and adaptability, being partly genetic (fixed) and partly shaped by the ecological context, including interspecific relationships. At the population level, habitat selection translates into the density of individuals in each habitat type, with densities higher and more stable in optimal habitats (Sundell et al. 2012). Some rodents (e.g., meadow voles) have been demonstrated to be habitat selectors, their densities reflecting active choice (Morris and MacEachern 2010). Marginal habitats are occupied when population densities are high and intraspecific competition decreases fitness in locations of optimal conditions (Rosenzweig 1981), resulting in density‐dependent habitat selection (Fretwell 1972; Rosenzweig 1981). However, habitat segregation may also result from species‐characteristic responses to habitat attributes (i.e., differences in preference—Sozio and Mortelliti 2016).

Habitat selection depends on the ecological context and may be organised spatially along major geographical gradients (Fortin et al. 2008). Plasticity of habitat use and selection at large spatial scales shows a three‐dimensional model along the longitudinal gradient (e.g., in red pandas—Bista et al. 2019), latitude (e.g., in woodland caribou—Fortin et al. 2008), and elevation (e.g., in spotted owls—Kramer et al. 2021). These patterns are dependent on intrinsic factors, i.e., differences in the physiological requirements of a species in different ecological (mostly climatic) contexts (Bista et al. 2019), and on extrinsic factors, such as resource availability (Kramer et al. 2021) and the presence or absence of competitors (Mandeville et al. 2024) or predation risk (Bhattacharyya et al. 2015; Gillingham and Parker 2008).

The yellow‐necked mouse, *Apodemus flavicollis* (Melchior, 1834), and the bank vole, *Clethrionomys glareolus* (Schreber, 1780), are the most abundant small mammal species in forest habitats across large parts of Europe and are very often syntopic (Amori et al. 2010; Benedek and Sîrbu 2019; Čepelka et al. 2020; Hille and Mortelliti 2011; Väli and Tõnisalu 2020; Viviano et al. 2022). Both are European species with mostly overlapping geographic distributions, except for the southern regions of *A. flavicollis* distribution and northern parts of *C. glareolus* distribution (Aulagnier et al. 2009), in relation to the somewhat more thermophilic nature of the mouse (Marsh et al. 2001) and the increase of the vole abundance at higher elevations and latitudes (Torre and Arrizabalaga 2008), showing its adaptation to cooler environments. Both are woodland habitat specialists (Sozio and Mortelliti 2016), inhabiting all types of forest and scrubby areas, hedges, orchards and parks (Aulagnier et al. 2009; Marsh et al. 2001; Torre and Arrizabalaga 2008). Their diet is less similar. *Apodemus flavicollis* is considered omnivorous (Aulagnier et al. 2009) or granivorous (Abt and Bock 1998), whereas *C. glareolus* is predominantly herbivorous (Abt and Bock 1998; Aulagnier et al. 2009). However, in forests, seeds are an important food for both species, and masting (tree fruiting) is an important driver of their population dynamics (Flowerdew et al. 2017; Selva et al. 2012; Zwolak et al. 2018), which results in a certain degree of exploitation competition (Amori et al. 2010; Buesching et al. 2008; Montgomery 1980). In addition to the differences in the diet, the two species show different diel activity patterns, resulting in temporal segregation, acting as another mechanism of coexistence. *Apodemus flavicollis* is strictly nocturnal (Aulagnier et al. 2009), with its activity peaking after midnight, and *C. glareolus* shows multiple activity peaks at dawn and dusk but also during the day (Canova 1993), which tend to intensify in the presence of *Apodemus* species (Viviano et al. 2022). When both *A. flavicollis* and *C. glareolus* are active, the bank vole tries to avoid areas where the mouse is present (Andrzejewski and Olszewski 1963; Greenwood 1978) as meetings between them were reported to be aggressive as a rule, with the yellow‐necked mice being the attackers and winners (Andrzejewski and Olszewski 1963). All these confirm the competitive interference between the two rodents and the dominance of *A. flavicollis* over *C. glareolus* , which is sustained by the larger size—its weight ranging between 22 and 56 g—compared to the bank vole—weighing between 15 and 40 g (Aulagnier et al. 2009), increased agility and aggressivity. The two species share a similar range of predators (Sundell et al. 2012), adding a component of apparent competition to their interaction. Still, these rodents show only slight ‘top‐down’ effects while undergoing strong ‘bottom‐up’ control (Flowerdew et al. 2017).

Despite the large number of studies on density‐dependent habitat selection in various spatial and ecological contexts, little is known about the combined effect of elevation and intra‐ and interspecific density differences. Studies on the interaction and habitat selection by *A. flavicollis* and *C. glareolus* were conducted before (Amori et al. 2015; Hille and Mortelliti 2011; Sozio and Mortelliti 2016), but none in the mountains, along the upper part of their elevational range, up to beyond the timberline, so our study seeks to fill this gap. The overall responses of small mammal assemblages to habitat attributes and human disturbance in South Carpathian forests were previously evaluated, and the strong divergence in the relative habitat use by the three most abundant species, namely the rodents *A. flavicollis* and *C. glareolus* and the shrew *Sorex araneus* Linnaeus, 1758, was interpreted as a potential mechanism enabling their coexistence as dominant species (Benedek et al. 2021). Here, we expand this approach and hypothesis by exploring the differential habitat selection by the two rodents along the elevational gradient and in various ecological contexts of conspecific and heterospecific density. We tested three hypotheses:

*Apart from differences in diet and activity patterns, the coexistence of these rodents in the relatively continuously forested landscape is facilitated by differences in habitat selection, resulting in a degree of habitat segregation. Therefore, we predict that most variation in abundance is explained by habitat characteristics that affect either species or both, but in opposite ways*.

*Because A. flavicollis is more thermophilous compared to C. glareolus and the climate is cooler at high compared to low elevations, we presume that these species have coexisted for a longer time in the lower parts of the mountains, where they had more time to differentiate their niches. Therefore, we hypothesise that habitat segregation between A. flavicollis and C. glareolus is stronger at low elevations, resulting in distinct (even opposite) models of their abundance in relation to habitat features. In contrast, at high elevations, we expect the two species to show more similar habitat selection models*.

*The two species exhibit an asymmetrical competition, with the larger and more agile mouse competitively superior. Therefore, we predict that the abundance of C. glareolus is negatively affected by the abundance of A. flavicollis but that the abundance of the mouse is unaffected by the abundance of the vole*.

## 2 Material and Methods

### 2.1 Study Area and Small Mammal Trapping

Our study was conducted during the warm months (end of June–September) between 2000 and 2007 in forests of the Southern Carpathian Mountains, Romania. Most sampling sites were located in the Retezat National Park and the others in the neighbouring Râul Șes river basin, both areas included in the ROSCI0217 Retezat, part of the Natura 2000 European network of protected areas. The study area is mostly covered by forests—a mixture of virgin, natural and planted. At the lowest elevation in the study area are the beech ( *Fagus sylvatica* ) forests, followed by mixed forests composed of differing proportions of beech and Norway spruce ( *Picea abies* ) with few other broadleaved tree species (e.g., *Acer pseudoplatanus, Abies alba, Sorbus aucuparia* and *Alnus incana* along the rivers). The cover, height and composition of the herbaceous layer in these forests depend on the tree canopy cover, moisture and mostly geological substratum, being richer on limestone compared to granitic rocks and schists as well as along the rivers, where the tall species prevail (e.g., *Petasites albus*, *P. hybridus* , *Adenostyles alliariae* and *Telekia speciosa*) and shrubs (e.g., *Sambucus racemosa, Rubus idaeus* and *Salix capraea*) are more common. The upper forest belt (mostly above 1300 m elevation) comprises almost exclusively spruce trees, and it reaches up to the timberline, which usually is present at elevations between 1600 and 1800 m a.s.l., depending on slope, exposition and other geomorphological characteristics of the site. The shrub and herbaceous layers in spruce forests are composed mainly of spruce saplings. Above the timberline, mugo pine ( *Pinus mugo* ) shrubs cover parts of the subalpine meadows.

We used artisanal wooden live box‐traps set in transects of 30–40 traps 15 m apart, along the contour lines and parallel to the closest watercourse, forest edge, road or trail, within homogeneous forest habitats. We set 81 transects in 43 trapping sites at elevations ranging between 820 and 2080 m. Below the timberline, all transects were set in forests. Some sites were on the river (or stream) banks; the others were at varying distances from them. Additional details on the research area and sampling design were previously published (Benedek et al. 2021).

All aspects of trapping and animal handling complied with EU Council Directive 86/609/EEC on experimental use of animals. Trapping within the protected area was done at the invitation of the Administration of Retezat National Park following the protocols on trapping and animal handling developed and approved by the Scientific Council of Retezat National Park. Subsequently, the working protocol was also approved by the Biomedical Research Ethics Commission of the Lucian Blaga University of Sibiu (approval 9/26 May 2021).

### 2.2 Habitat Variables

For each trap line, we evaluated several habitat variables, which were previously found to be important factors of habitat selection in rodents (e.g., Lehtonen et al. 2001; Naxara et al. 2009; Suárez‐Gracida and Álvarez‐Castañeda 2009), included as predictors in the regression models. As vegetation characteristics, we evaluated the mean height of the herbaceous layer (in cm) (HHeight), the percent cover of herbaceous (HCov) and shrub (SCov) layers and of the tree canopy (TCov), also recorded separately for coniferous trees (Con). We estimated tree cover (TCov) by the percent of the ground where the light fell directly and also measured the distances from the centre point of each transect to the closest watercourse, river or creek (in km) (DWat) and the elevation (in m) (Elev). Estimates of soil moisture (Moist), abundance of rocks (Rocks) and coarse woody debris (Logs) were included in analyses as ordinal variables. Moisture was evaluated based on the composition and structure of the vegetation and ordered as: 1—xeric, 2—mesic, 3—meso‐hydric and 4—hydric. Levels of Logs and Rocks were: 0—absent, 1—isolated, 2—scarce, 3—moderate and 4—abundant. The overall descriptive statistics of these environmental variables were previously reported (Benedek et al. 2021). Here, we present their descriptive statistics separately for low and high elevations (Table A1).

### 2.3 Data Analysis

To account for the large differences in the variation of predictors and obtain comparable (standardised) regression coefficients, we applied the standard normalisation, centering and rescaling of the variables to have the mean of 0 and the standard deviation of 1 (*z*‐scores). To test for the multicollinearity of predictors, we constructed a multiple non‐parametric (Spearman) correlation matrix of predictors in package *Hmisc* (Harrell 2023). The correlation coefficients were ≤ 0.6, a value lower than those considered to indicate potential collinearity problems in multiple regression models (Dormann et al. 2013). Therefore, we kept all the habitat variables in the analyses. In addition, for all the best models, we calculated the variance inflation factor (VIF) for the included predictors, and all VIF values were < 5, confirming the lack of multicollinearity (Akinwande et al. 2015).

Because our aim was to evaluate the differential habitat selection in relation to elevation and population density, we split the dataset in two. Firstly, based on elevation, we got two datasets, for LOW and HIGH. LOW included transects (*n* = 42) below or at 1300 m a.s.l. and HIGH (*n* = 39) transects above this elevation. As mentioned in the description of the area, above 1300 m (as an average because there are local variations both natural—climatic inversions—and artificial—plantations of spruce in clearings of former mixed or beech forests) forests are dominated by spruce, the typical high‐elevation forests. In addition, this is close to the middle of the elevational gradient in our study, resulting in balanced (similarly‐sized) elevational samples. Secondly, based on the year of survey, we got another two datasets, ODD (survey years 2003, 2005, 2007; *n* = 50) and EVEN (2000, 2002, 2004, 2006; *n* = 31) years, characterised by alternately low and high population densities of the two rodents. To test the differences in the abundance of the two species between LOW and HIGH and ODD and EVEN, we used the non‐parametric test for two independent samples (Wilcoxon rank sum test).

To evaluate habitat selection in relation to elevation and population density, for each subset, we constructed generalised linear mixed models (GLMM) built in package *lme4* (Bates et al. 2015), including as response variables the relative abundance of *A. flavicollis* and *C. glareolus* , expressed as trapping rate, i.e., the number of individuals (excluding recaptures)/100 trap‐nights, as a proxy for local population density and thus a measure of habitat use (Sundell et al. 2012). The predictors were the habitat characteristics. In the regression models, the coefficients are the measures of selection. Positive coefficients show preferential use, negative ones show avoidance, whereas non‐significant coefficients indicate random exploitation of those resources (Dueser and Hallett 1980). We included year as a random factor to account for the lack of independence of data from the same year (only random intercepts models). Because the trapping effort varied among transects, it was included in the models as offset. We tested overdispersion in package *performance* (Lüdecke et al. 2021), and if significant, we constructed negative binomial GLMMs; otherwise, we used Poisson GLMMs. Because in ODD, most of the transects had no *A. flavicollis* , resulting in a large number of 0's in the response variable (44—for the transects without the mouse), we tested the excess of 0's (zero‐inflation) using the function check_zeroinflation in package *performance*, but it was not significant. Interannual variations were also not significant, so the year was not included as a random factor. Therefore, for the yellow‐necked mouse in ODD, we report the results of the Poisson GLM.

To quantify habitat selection intensity, we used the amount of variation in the abundance explained by the habitat features, expressed by partial *r* ^2^, which gives the proportion of variation explained by the predictors in the full model that cannot be explained by the explanatory variables in the reduced model that does not include these. To evaluate habitat segregation (H1), we selected the models with ΔAICc < 2 compared to the best models using the *MuMIn* package (Bartoń 2023). However, in both species, the competing models (ΔAICc > 0) included variables with non‐significant effects (*p* > 0.05). Therefore, instead of averaged models, we compared and reported the best models, which include only significant predictors. The significance of predictors was evaluated using the likelihood‐ratio test (LR test). We partitioned variation explained by the contrasting habitat features (those with significant but opposite effects in the two species) and those with similar effects using the *partR2* package (Stoffel et al. 2021). We quantified the dissimilarity between the habitat selection by the mouse and vole (H2) as the Euclidean distance between their position in the space defined by the significant habitat features given by the averaged regression coefficients of the subset of best competing models (ΔAICc < 2), weighted by their partial *r* ^2^. To test the significance between the distances at low and high elevations, we calculated the 95% confidence intervals based on jackknife resampling, removing in turn each of the 1638 combinations of transects in LOW and HIGH elevations. To test the competition hypothesis (H3), we evaluated the effect of the competitor by including it in the habitat selection model (Dueser and Hallett 1980), also testing the interaction between habitat attributes and the competitor abundance. All analyses were performed in R version 4.3.0 (R Core Development Team 2023).

## 3 Results

**FIGURE 1:**
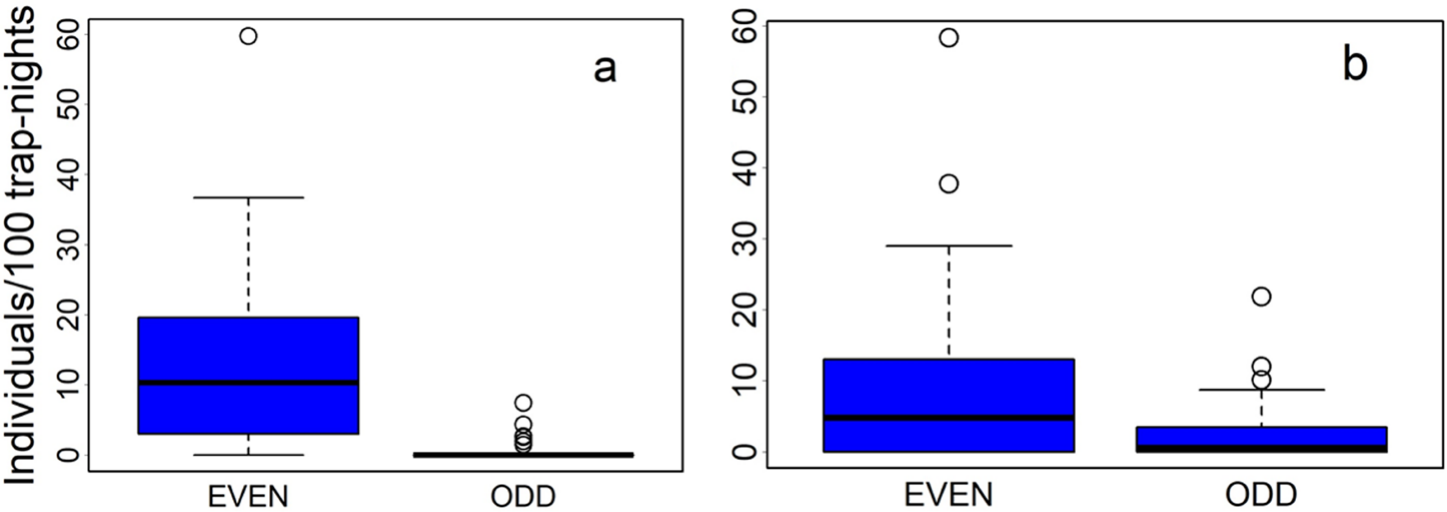
Abundance of (a) *A. flavicollis* and (b) *C. glareolus* in high‐ (EVEN) and low‐density (ODD) years.

**FIGURE A1:**
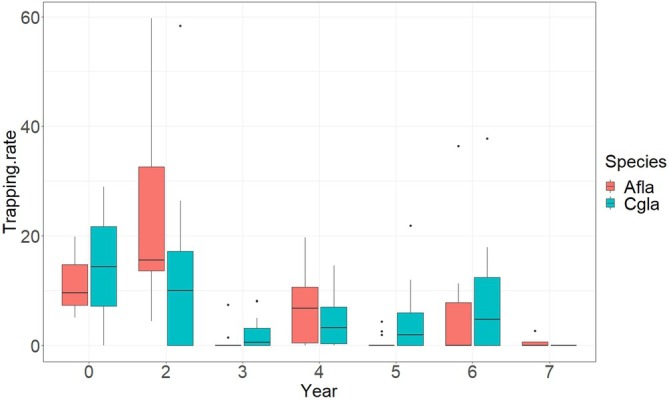
Trapping rates (indiviuals/100 trap‐nights) of the yellow‐necked mouse (Afla) and bank vole (Cgla) in the survey years (represented by the number of years after 2000).

*Apodemus flavicollis* (220 individuals captured in 30 transects) and *C. glareolus* (206 individuals in 45 transects) represented 78.7% of the total captures (541 individuals of 12 species). Their abundance fluctuated significantly from year to year (Figure A1), with lower densities in ODD years (Wilcoxon *W* = 1343, *p* < 0.001 for *A. flavicollis* —Figure 1a and Wilcoxon *W* = 1027.5, *p* = 0.010 for *C. glareolus* —Figure 1b), but not with elevation.

**FIGURE 2:**
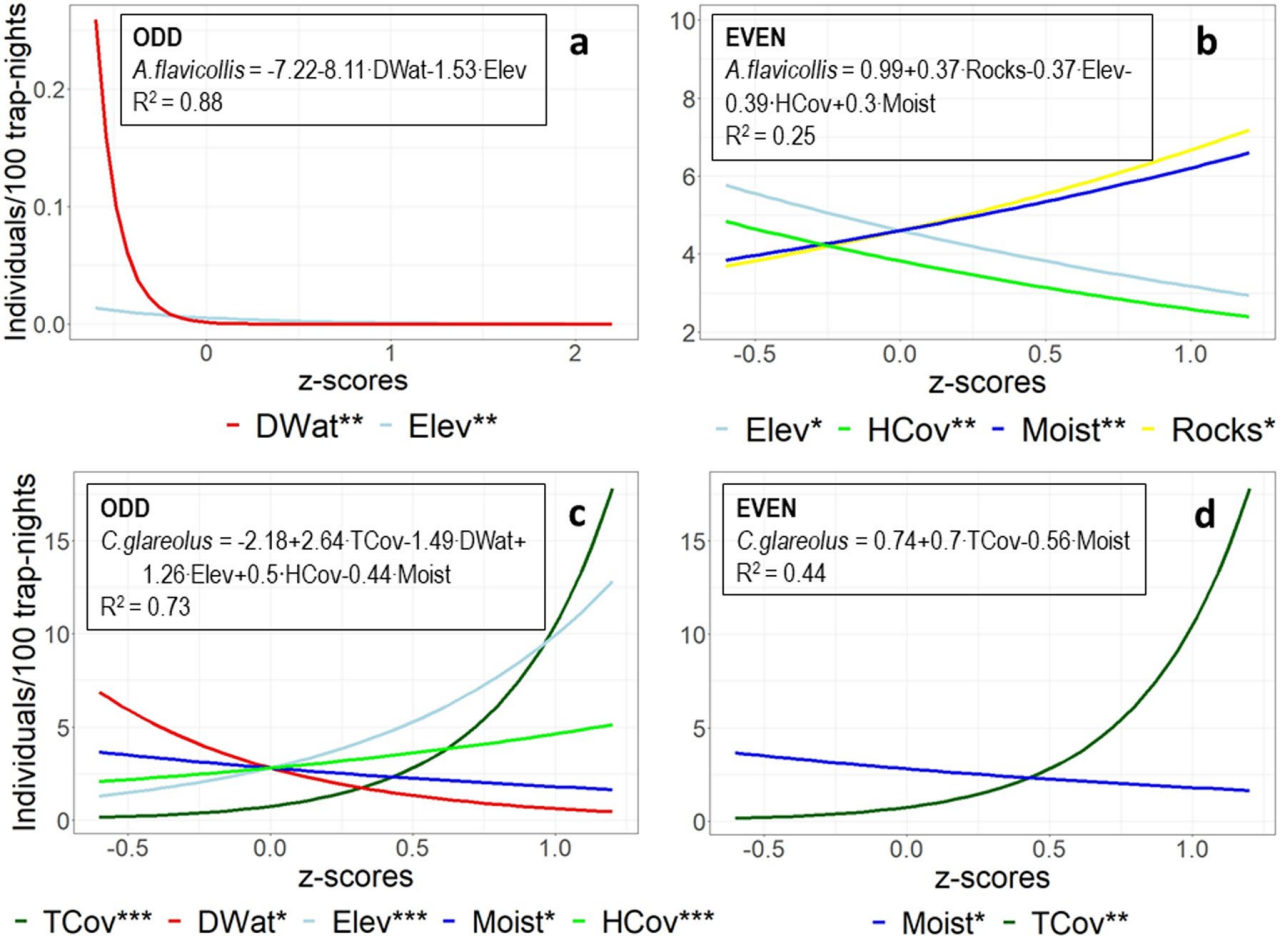
Response curves for *A. flavicollis* in (a) ODD and (b) EVEN years and *C. glareolus* in (c) ODD and (d) EVEN years. Habitat variables are standardised, and the overlapping range of their *z*‐scores is presented. For each habitat variable, the predicted values are calculated for the years 2005 (in ODD) and 2004 (in EVEN), for a trapping effort of 100 trap‐nights and for the mean value (*z*‐score = 0) of the other variables. The asterisks in the legends represent the significance of the predictors: *0.01 < *p* < 0.05, **0.001 < *p* < 0.01, ***p < 0.001. DWat—distances from the centre point of each transect to the closest watercourse, river or creek (in km); Elev—elevation (in m); HCov—percent cover of the herbaceous layer; Moist—ordinal variable expressing the soil moisture; Rocks—ordinal variable expressing the abundance of rocks; TCov—percent cover of the tree canopy.

In ODD years, *A. flavicollis* was seldom captured (seven individuals in six transects), at low elevations (at maximum 1120 m a.s.l) close to the river banks. These two variables accounted for 88.6% of variation in its abundance (Figure 2a, Table A2). In EVEN years, the mouse was still more abundant at lower elevations and showed a negative response to HCov and positive response to Moist and Rocks (Figure 2b). The significant variables accounted for 25.3% of the variation in abundance, the 95% confidence interval being contingent with that of the model for low population density years (Table A2).

In ODD years, *C. glareolus* was more abundant at higher elevations far from watercourses and showed a positive response to TCov and HCov and a negative response to Moist (Figure 2c), which together explained 73.5% of the variation in its abundance (Table A3). In EVEN years, the vole was still more abundant in dry habitats with high tree cover. These variables accounted for only 44.4% of the variation in abundance (Figure 2d), and the 95% confidence intervals of the explained variation in ODD and EVEN years overlapped (Table A3).

### 3.1 Test of H1. Habitat Segregation Facilitates the Coexistence of the Two Rodents

**FIGURE 3:**
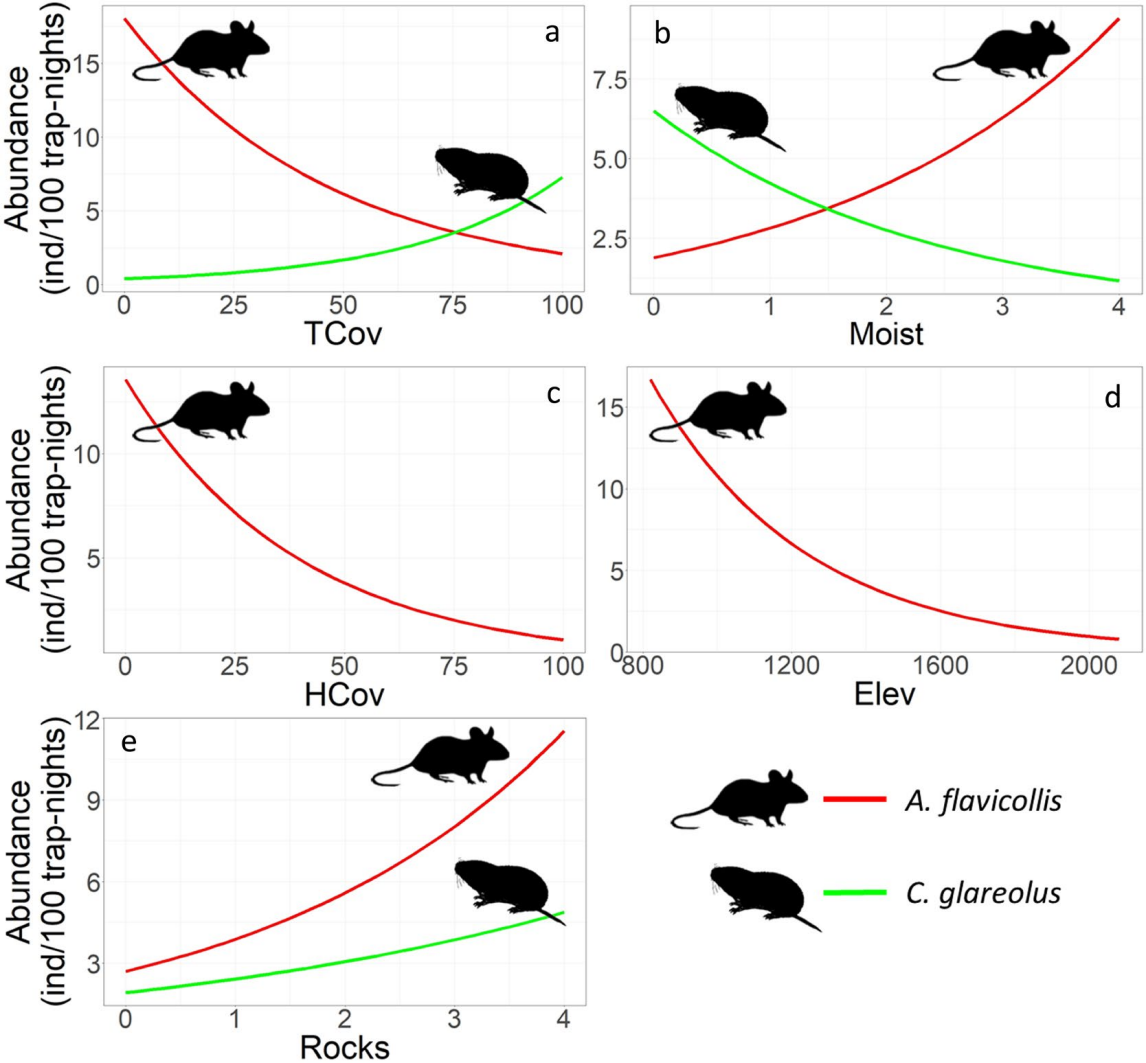
Curves of responses of *Apodemus flavicollis* and *Clethrionomys glareolus* to habitat attributes with opposite effects: (a) tree cover (TCov), (b) moisture (Moist), effects on one species: (c) herbaceous cover (HCov), (d) elevation (Elev), and consistent effects on both species: (e) abundance of rocks (Rocks). For each habitat variable, the predicted values are calculated for the year 2004, for a trapping effort of 100 trap‐nights, and for the mean value of the other variables.

The overall models included five predictors for *A. flavicollis* and three for *C. glareolus* . TCov and Moist had opposite effects, with the mouse being more abundant in moist habitats with sparse tree cover and the vole in dry compact forests (Figure 3a,b, Table 1). HCov and Elev had significant effects only on the mouse, which was more abundant at low elevations in habitats with sparse herbaceous cover (Figure 3c,d, Table 1). The contrasting variables (affecting either one species or both, but in opposite ways) accounted for most of the explained variation—63% (7.5% of the total 11.9% explained variation) in the mouse and 93.6% (35% of the total 37.4%) in the vole. Both species favoured rocks (Figure 3e, Table 1), but these accounted for only 4.4% and 2.4% of the explained variation in the abundance of the mouse and vole.

**TABLE 1:** Coefficients and standard errors of the best models explaining the abundance (expressed in the number of captured individuals per 100 trap‐nights) of the two rodent species, *Apodemus flavicollis* and *Clethrionomys glareolus,* by their habitat characteristics.

Variable	*Apodemus flavicollis*			*Clethrionomys glareolus*		
	Coef. ± SE	*χ* ^2^	*p*	Coef. ± SE	*χ* ^2^	*p*
TCov	**−0.62 ± 0.14**	20.69	< 0.001	**1.12 ± 0.2**	27.6	< 0.001
Moist	**0.44 ± 0.09**	23.5	< 0.001	**−0.49 ± 0.17**	7.7	0.005
HCov	**−0.73 ± 0.11**	40.2	< 0.001	—	—	—
Elev	**−0.77 ± 0.15**	29.9	< 0.001	—	—	—
Rocks	0.42 ± 0.13	10.7	0.001	0.43 ± 0.17	6.5	0.01
Model *r* ^2^ (95% CI)	0.12 (0.07, 0.48)			0.37 (0.2, 0.61)		
Contrasting *r* ^2^ (95% CI)	**0.08 (0.04, 0.38)**			**0.35 (0.18, 0.59)**		

*Note:* The codes of habitat variables are explained in the text. In bold are coefficients and *r* ^2^ (with 95% confidence interval) for contrasting predictors (i.e., with effects on one species or opposite effects on both species).

### 3.2 Test of H2. The Habitat Segregation Between *Apodemus flavicollis* and *Clethrionomys glareolus* is Weaker at High Elevations

**FIGURE 4:**
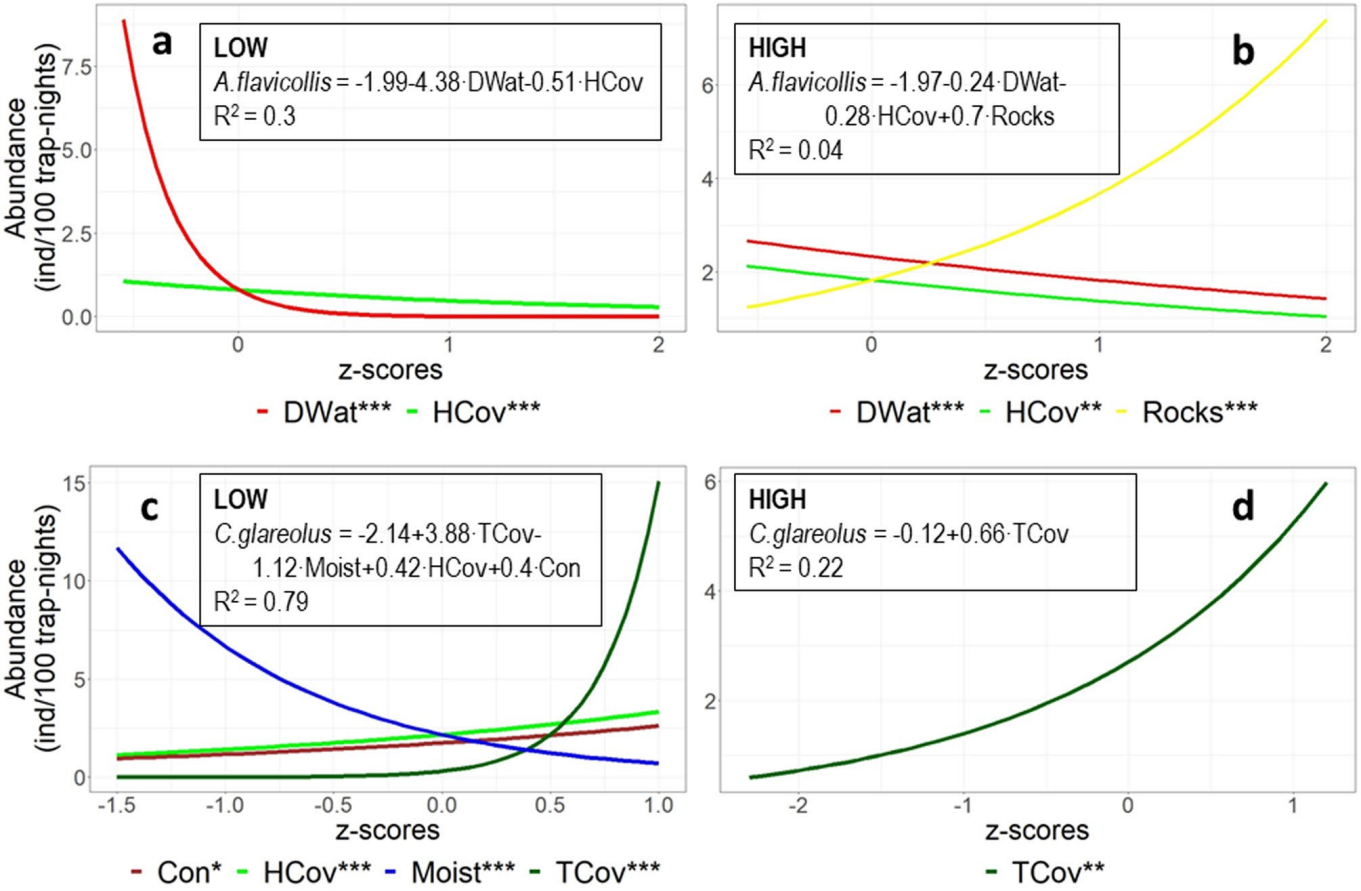
Response curves for *Apodemus flavicollis* at (a) LOW and (b) HIGH elevations and *Clethionomys glareolus* at (c) LOW and (d) HIGH elevations. Habitat variables are standardized, and the overlapping range of their *z*‐scores is presented. For each habitat variable, the predicted values are calculated for 2004, for a trapping effort of 100 trap‐nights and for the mean value (*z*‐score = 0) of the other variables. The asterisks in the legends represent the significance of the predictors: * 0.01 < *p* < 0.05, ** 0.001 < *p* < 0.01, ** *p* < 0.001. Con—percent cover of coniferous trees within the canopy; DWat—distances from the transect to the closest watercourse, river or creek (in km); HCov—percent cover of the herbaceous layer; Moist—ordinal variable expressing the soil moisture; Rocks—ordinal variable expressing the abundance of rocks; TCov—percent cover of tree canopy.

In the LOW elevation model, *A. flavicollis* showed highly significant negative responses to DWat and HCov, which explained 30.1% of the variation in its abundance (Figure 4a). In the HIGH elevation model, the mouse was still more abundant close to watercourses and showed a negative response to HCov and a positive response to Rocks (Figure 4b). However, these variables had low explanatory power, accounting for only 3.9% of the variation in abundance, and the 95% confidence interval largely overlapped with that of the model for LOW elevation (Table A4).

In the LOW elevation model, *C. glareolus* had a positive response to Con, TCov and HCov and a negative response to Moist (Figure 4c), which together had very good explanatory power, accounting for 79.5% of the variation in its abundance (Table A5). In contrast, in the HIGH elevation model, TCov was the only significant predictor (Figure 4d), explaining only 22.5% of the variation in abundance. In contrast with the mouse, the 95% confidence intervals of the explained variation in the vole abundance at LOW and HIGH elevations did not overlap (Table A5).

**FIGURE 5:**
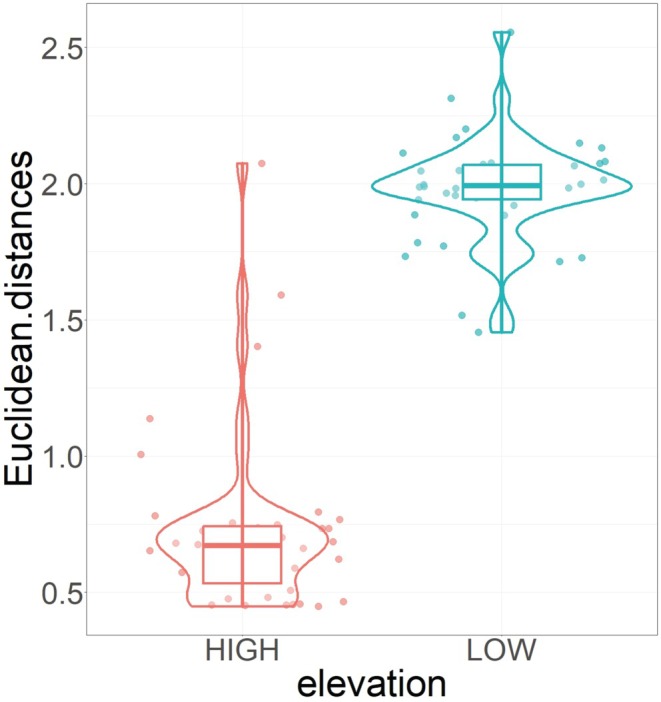
Violin plot illustrating the distribution of Euclidean distances between the responses of *Apodemus flavicollis* and *Clethrionomys glareolus* to habitat attributes at low (LOW, ≤ 1300 m a.s.l.) and high (HIGH, > 1300 m a.s.l.) elevations.

Based on the averaged standardized regression coefficients of the best competing models explaining the abundance of the two species at LOW and HIGH elevations (Table 2), we calculated a Euclidean distance of 1.51 for LOW and 1.07 for HIGH elevations. The mean distance of the 1638 jackknife samples was 1.98 (95% CI: 1.92, 2.04) for LOW elevation and 0.72 (95% CI: 0.62, 0.83) for HIGH elevation (Figure 5), the difference being significantly larger than 0 (*t* = 135.9, df = 1637, *p* < 0.001), with a mean of 1.25 (95% CI: 1.23, 1.27).

**TABLE 2:** Averaged standardised regression coefficients (and their standard errors) of the best competing models (ΔAIC < 2) explaining the standardised abundance (*z*‐scores of the number of captured individuals per 100 trap‐nights) of *Apodemus flavicollis* and *Clethrionomys glareolus* at LOW and HIGH elevations.

Variable	LOW		HIGH	
	*A. flavicollis*	*C. glareolus*	*A. flavicollis*	*C. glareolus*
DWat	−3.76 ± 1.53 (8.2)	0	−0.18 ± 0.14 (4.2)	0.61 ± 0.37 (1.3)
HCov	−0.64 ± 0.2 (4.9)	0.38 ± 0.12 (2.5)	−0.13 ± 0.5 (2)	0
Rocks	0	0	0.54 ± 0.21 (< 1)	0.59 ± 0.35 (6.3)
Con	0	0.19 ± 0.23 (< 1)	1.4 ± 1.54 (1.6)	1.52 ± 0.52 (34.6)
TCov	0	2.98 ± 0.75 (69.4)	−0.72 ± 1.17 (1.2)	0.93 ± 0.35 (33.5)
Moist	0.54 ± 0.32 (3.1)	−1.07 ± 0.17 (4.1)	0.40 ± 0.15 (2)	−0.61 ± 0.36 (< 1)
SCov	0	0	−0.73 ± 0.51 (2.1)	−0.96 ± 0.56 (1.9)
HHeight	0	0	−0.32 ± 0.26 (< 1)	1.3 ± 0.73 (< 1)
Logs	0	0.05 ± 0.12 (1.2)	−0.6 ± 0.36 (< 1)	0

*Note:* The codes of habitat variables are given in the text. The values in the parentheses are the partial *r* ^2^ used to weigh the coefficients when calculating the Euclidean distances between the species' responses to habitat features.

### 3.3 Test of H3. The Two Rodents Exhibit Asymmetrical Competition, With *Apodemus flavicollis* Being the Stronger Competitor

In the model relating the abundance of *A. flavicollis* as the response variable to the significant habitat attributes and the abundance of its competitor *C. glareolus* as predictors, the vole had a negative (*b* = −0.16, SE = 0.07) and significant (*χ* ^2^ = 6.12, df = 1, *p* = 0.013) effect on the mouse, accounting for 12.5% of the explained variation, but the effect of the interactions between competitor abundance and habitat attributes was not significant. In contrast, in the model with the vole abundance as a response to the abundance of the mouse in addition to habitat attributes, the mouse abundance had no significant effect.

## 4 Discussion

Here, we evaluate the differential population‐level habitat selection by *A. flavicollis* and *C. glareolus* along an elevational gradient in Southern Carpathian mountain forests, where their abundance fluctuates strongly among years, with peak years followed by low‐density years (Benedek and Sîrbu 2019). Overall, the variation in abundance explained by the habitat features (the intensity of habitat selection) was lower for *A. flavicollis* than for *C. glareolus* , suggesting the more specialised nature of the vole. In ODD years, when density was low, both species showed a high degree of specialisation. However, the difference was significant only for the mouse (Table A1), thus demonstrating a density‐dependent habitat selection, which has been documented for various taxa, including small mammals, e.g., desert rodents (Shenbrot 2004), voles (Morris and MacEachern 2010; Sundell et al. 2012) and lemmings (Morris and Dupuch 2012).

In accordance with our expectations, most of the variation in the overall abundance of the two rodents was explained by the contrasting variables (H1). The two species showed different elevational patterns, with the mouse abundance decreasing along the elevational gradient and the vole showing no significant pattern, in accordance with their different thermal tolerances (Marsh et al. 2001; Torre and Arrizabalaga 2008). *Clethrionomys glareolus* selected dry habitats with dense canopy cover. In contrast, *A. flavicollis* selected moist habitats with less closed canopy and, in addition, sparse herbaceous cover. The only habitat feature consistently selected by both species was the abundance of rocks. Rocks are stable elements of the habitat that provide refuge and protection for nests, enhancing the survival of adults and young. The large variation in abundance explained by the contrasting predictors indicates a significant habitat segregation that may result from competition, constituting not only a mechanism of coexistence but also of codominance. Although habitat segregation is usually stronger among morphologically similar, related species (Naxara et al. 2009; Suárez and Bonaventura 2001), our results show that spatial segregation, in the form of habitat overspecialisation, may act as a coexistence mechanism for these more distantly related and morphologically different species.

The responses of the two rodents to habitat attributes were consistent at low and high elevations. *Clethrionomys glareolus* showed a significantly more generalist response at high elevations (only 22% of the variation in abundance explained by habitat attributes), where it responded only to tree cover, indicating that dense forests at high elevations are its optimal habitat. This contrasts with other studies, which mention riverine lowland forests as the optimal habitat of *C. glareolus* (Aulagnier et al. 2009), suggesting a larger variation in habitat selection. At low elevations, where habitat attributes explained 79% of the variation in its abundance, the effect of tree cover was stronger and *C. glareolus* was also favoured by the cover of herbaceous vegetation and the proportion of conifers in the canopy. The latter may be partly a confounding effect with the elevation because elevation was not included in the construction of this set of models. Moisture had a negative effect, with abundances higher in drier habitats, contrasting with results in other parts of Europe (Aulagnier et al. 2009) and also from neighbouring lowland (Benedek and Sîrbu 2018). This difference in the response to moisture is probably related to the nature of the bedrock. The geological substratum of the study area includes acidic and basic rocks, and the soils of the latter are drier and support much more lush and diverse vegetation (Benedek et al. 2004), favouring the herbivorous vole.

In contrast to *C. glareolus* , *A. flavicollis* appeared to be a generalist both at low and high elevations. Because of its more thermophilous nature compared to the vole, one might expect more intense habitat selection by the mouse at high elevations. The lack of the expected selection might be related to the fact that here the mouse was captured only in high‐density years, when probably many individuals were dispersing. This indicates the need for a larger trapping effort in low‐density years, which would also allow establishing whether *A. flavicollis* is still present at high elevations, at very low densities that make it difficult to detect, or it is mostly absent, recolonising higher elevations when densities increase, probably by dispersing along valleys. The latter possibility may explain the strong negative effect of the distance to the closest watercourse in low‐density years (Table A2).

As expected, the distance between the responses of *A. flavicollis* and *C. glareolus* to habitat characteristics was smaller at high elevations (H2). This elevational difference conforms to the expectation of habitat segregation as a coexistence mechanism of the two species. Because the mouse has probably only recently reached high elevations, where its occurrence is still ephemeral, limited to high‐density years, the competition with the vole is less intense there, hence the weaker habitat segregation. This may be the effect of past (Connell 1980) and present competition (Begon et al. 2009), resulting in the diminution of the latter and promoting coexistence. Although habitat segregation may also be the result of differential physiological and behavioural adaptations to environmental conditions experienced in allopatry (Biedma et al. 2020), the elevational differences between the habitat selection patterns in the two species in our study suggest that at least part of their habitat segregation results from competition.

In the habitat selection model, when the abundance of the putative competitor is included with a negative coefficient, we may infer a competitive interference (Dueser and Hallett 1980). When only one of the models for the two species includes the competitor as having a significant negative effect on its abundance, we may infer asymmetrical competition between the two (H3). A significant interaction between the competitor and habitat features may point to a competitive effect on selected habitat attributes. Contrary to expectations, *C. glareolus* was not affected by the abundance of *A. flavicollis* , whereas the mouse was negatively affected by the vole. This reflects asymmetric competition between the two rodents, with the mouse, contrary to expectations based on its larger size, higher agility and aggresivity, appearing to be the weaker competitor. Studies that show *A. flavicollis* to be a stronger competitor than *C. glareolus* (Andrzejewski and Olszewski 1963; Viviano et al. 2022; Wójcik and Wołk 1985) come from lower elevations, from habitats closer to the ecological optimum of the mouse. Our study area is closer to the optimum habitat of the vole; hence, the contrasting results confirm that towards the pessimum, competitive abilities decrease even in strong competitors. Condition‐specific competition, where the outcome of the competitive interaction depends on the features of the environment, is well documented along the elevational gradient in various taxa (Lyu and Alexander 2022; Mandeville et al. 2024; Sirén and Morelli 2020). In addition, habitat selection was stronger in *C. glareolus* , making it a habitat specialist in comparison with the more generalist *A. flavicollis*, and in their specific habitats, specialists are usually dominant in the competitive relationship (Biedma et al. 2020). In our study, we missed some habitat features (e.g., soil characteristics) that may be important for rodents, and we cannot completely exclude that the negative effect of the vole on the mouse may have been produced also by the correlation with the unmodelled habitat variables. However, negative effects of *C. glareolus* on *A. flavicollis* were also recorded in other studies, e.g., in the Mediterranean region (central Italy), but there, the effects were symmetrical, and the survival, recruitment and body mass effects were expressed at the individual level and not reflected at the population level (Sozio and Mortelliti 2016).

Habitat selection is a hierarchical process that takes place at different scales (Johnson 1980; Jorgensen 2004; Mayor et al. 2009), and the spatial scale of the research has a strong influence on its results. Therefore, expanding this analysis to other spatial levels, both higher—landscape (Fortin et al. 2008)—and lower—microhabitat (Amori et al. 2015; Hille and Mortelliti 2011; Jorgensen 2004)—could provide insight into the hierarchical process of habitat selection and prediction power of the effects of natural or anthropic changes in the environment on animal populations, enabling the development of effective conservation and management strategies (Villanueva‐Hernández et al. 2017). Replicating this study over elevational gradients in other European mountain areas could test the generality of our results. The differences in latitude and past and present forest management practices are probably the drivers of the peculiarities of the overall habitat use in this area compared to that reported from Northern Carpathians (Benedek et al. 2021). Therefore, these may also influence the elevational and density‐dependent patterns of habitat selection. Because the simple presence of an animal does not always reflect habitat selection (Beyer et al. 2010), further research should consider only resident individuals who actively select the habitat, whose features affect their fitness. An alternative would be to look at the presence/absence data of the two rodents combined with data on the population (age) structure and biometric (body condition index) information. These could provide deeper insight into the patterns of habitat selection by these species and help decipher the mechanisms that govern it. In addition, processes such as limited perception of risks (resulting in ecological traps), social structure and despotic behaviour (Kelt et al. 2019), site familiarity or time lags (Kawaguchi and Desrochers 2018), which may interfere with habitat selection (Boyce et al. 2016) sometimes resulting in the misreflection of habitat quality (Kelt et al. 2019), should also be considered.

Although concerns have been raised on statistical methods applied to observational studies on habitat and competition, which provide only equivocal means of separating their confounding effects (Rosenzweig and Abramsky 1985), statistical modelling of observational data allows for exploring competitive relationships on large scales, answering questions that are very difficult to address experimentally (Pimm 1985), such as, in our case, their change along the elevational gradient. Our study provides insight into the differential habitat selection and the relationship between *C. glareolus* and *A. flavicollis* in the mountains, showing that habitat segregation is probably one of the mechanisms allowing for the coexistence of the two dominant rodents in the rather continuously forested landscape. The results also suggest asymmetrical competition between the two species and a possible recent elevational expansion of the more thermophilous mouse. Elevational patterns of habitat selection have implications for the climate change effect (Mandeville et al. 2024), and studying them is especially important in the case of keystone species, such as the rodents that prevail within terrestrial mammal communities because their dynamics induced by the changes in temperature and rainfall regime will result in changes in the functioning of montane ecosystems.

## Author Contributions

**Ana Maria Benedek:** conceptualization (lead), data curation (lead), formal analysis (lead), funding acquisition (lead), investigation (lead), methodology (lead), visualization (lead), writing – original draft (lead), writing – review and editing (equal). **Ioan Sîrbu:** conceptualization (supporting), data curation (supporting), formal analysis (supporting), investigation (supporting), methodology (supporting), visualization (supporting), writing – original draft (supporting), writing – review and editing (equal).

## Conflicts of Interest

The authors declare no conflicts of interest.

## Data Availability

The raw data used for this study are publicly available in the Dryad data repository. Benedek, A. M., Sîrbu, I., and Lazăr, A. Small mammals and their environment in Romania. Dryad https://doi.org/10.5061/dryad.zw3r2287s (2021).

## Appendix A

**TABLE A1:** Descriptive parameters of the habitat variables at low (LOW) and high (HIGH) elevations.

Parameter	DWat	TCov	SCov	HCov	HH	Con	Rocks	Logs	Moist	Elev
LOW										
Minimum	0	0	0	0	0	0	0	0	0	820
Maximum	0.2	100	70	100	75	100	4	4	4	1300
Median	0.05	80	10	40	30	55	1	1	2	1165
Mean	0.045	75.6	16.67	41.55	31.79	42.62	0.952	1.143	1.881	1115
95% CI lower	0.029	69.29	11.02	33	26.26	31.52	0.61	0.85	1.6	1066.9
95% CI upper	0.06	81.89	22.32	50.09	37.31	53.72	1.29	1.44	2.16	1163.5
HIGH										
Minimum	0	0	0	0	0	0	0	0	1	1320
Maximum	1.1	90	60	100	50	100	4	3	4	2080
Median	0.1	70	10	40	30	100	2	1	2	1550
Mean	0.191	54.36	17.31	46.92	25.51	76.67	1.769	1.282	2.359	1642
95% CI lower	0.103	43.82	12.43	37.27	22.78	62.87	1.42	0.97	1.96	1577.6
95% CI upper	0.278	64.89	22.18	56.57	28.24	90.47	2.11	1.6	2.76	1705.9

*Note:* Codes of habitat variables are given in the text.

Abbreviation: 95% CI lower, 95% CI upper—lower and upper limits of the 95% confidence interval of the mean.

**TABLE A2:** Parameters of the best models explaining the abundance (expressed in number of captured individuals per 100 trap‐nights) of *Apodemus flavicollis* in years of low (ODD) and high (EVEN) population density.

ODD				EVEN			
Variable	Coef. ± SE	*χ* ^2^	*p*	Variable	Coef. ± SE	*χ* ^2^	*p*
Elev	−1.53 ± 0.76	6.93	0.008	Elev	−0.37 ± 0.14	5.99	0.014
DWat	−8.11 ± 4.31	8.58	0.003	HCov	−0.39 ± 0.12	9.21	0.002
				Moist	0.3 ± 0.11	6.04	0.014
				Rocks	0.37 ± 0.15	5.02	0.025
Model significance		< 0.001		Model significance		< 0.001	
Marginal *r* ^2^ (CI)		0.88 (0.66, 0.99)		Marginal *r* ^2^ (CI)		0.25 (0.11, 0.64)	
Conditional *r* ^2^ (CI)		0.88 (0.74, 0.99)		Conditional *r* ^2^ (CI)		0.82 (0.53, 0.94)	

*Note:* Codes of habitat variables are given in the text.

Abbreviations: CI—95% confidence interval of the *r* ^2^ coefficient of determination; Coef. ± SE—value of the regression coefficient and its standard error.

**TABLE A3:** Parameters of the best models explaining the abundance (expressed in number of captured individuals per 100 trap‐nights) of *Clethrionomys glareolus* in years of low (ODD) and high (EVEN) population density.

ODD				EVEN			
TCov	2.64 ± 0.49	57.53	< 0.001	TCov	0.7 ± 0.22	7.59	0.005
Moist	−0.44 ± 0.19	5.47	0.019	Moist	−0.57 ± 0.24	5.1	0.023
HCov	0.5 ± 0.14	10.9	< 0.001				
DWat	−1.49 ± 0.6	7.89	0.005				
Elev	1.26 ± 0.31	22.05	0.001				
Model significance		< 0.001		Model significance		0.004	
Marginal *r* ^2^ (CI)		0.73 (0.46, 0.93)		Marginal *r* ^2^ (CI)		0.44 (0.23 0.74)	
Conditional *r* ^2^ (CI)		0.89 (0.69, 0.96)		Conditional *r* ^2^ (CI)		0.46 (0.21, 0.79)	

*Note:* Codes of habitat variables are given in the text.

Abbreviations: CI—95% confidence interval of the *r* ^2^ coefficient of determination; Coef. ± SE—value of the regression coefficient and its standard error.

**TABLE A4:** Parameters of the best models explaining the abundance (expressed in number of captured individuals per 100 trap‐nights) of *Apodemus flavicollis* in years of low (LOW) and high (HIGH) elevations.

LOW				HIGH			
Variable	Coef. ± SE	*χ* ^2^	*p*	Variable	Coef. ± SE	*χ* ^2^	*p*
DWat	−4.38 ± 1.1	20.77	< 0.001	DWat	−0.24 ± 0.07	11.1	< 0.001
HCov	−0.51 ± 0.12	18.44	< 0.001	HCov	−0.28 ± 0.1	7.43	0.006
				Rocks	0.7 ± 0.17	18.2	< 0.001
Model significance		< 0.001		Model significance		< 0.001	
Marginal *r* ^2^ (CI)		0.3 (0.17, 0.63)		Marginal *r* ^2^ (CI)		0.04 (0.01, 0.38)	
Conditional *r* ^2^ (CI)		0.92 (0.73, 0.99)		Conditional *r* ^2^ (CI)		0.97 (0.48, 1)	

*Note:* Codes of habitat variables are given in the text. Elev was not included in these analyses.

Abbreviations: CI—95% confidence interval of the *r* ^2^ coefficient of determination; Coef. ± SE—value of the regression coefficient and its standard error.

**TABLE A5:** Parameters of the best models explaining the abundance (expressed in number of captured individuals per 100 trap‐nights) of *Clethrionomys glareolus* at low (LOW) and high (HIGH) elevations.

LOW				HIGH			
TCov	3.88 ± 0.71	53.84	< 0.001	TCov	0.66 ± 0.24	5.03	0.024
Moist	−1.12 ± 0.16	49.89	< 0.001				
Con	0.4 ± 0.19	4.5	0.033				
HCov	0.42 ± 0.11	14.43	< 0.001				
Model significance		< 0.001		Model significance		0.024	
Marginal *r* ^2^ (CI)		0.79 (0.63, 0.93)		Marginal *r* ^2^ (CI)		0.22 (0.05, 0.52)	
Conditional *r* ^2^ (CI)		0.97 (0.93, 0.99)		Conditional *r* ^2^ (CI)		0.33 (0.07, 0.62)	

*Note:* Codes of habitat variables are given in the text. Elev was not included in these analyses.

Abbreviations: CI—95% confidence interval of the *r* ^2^ coefficient of determination; Coef. ± SE—value of the regression coefficient and its standard error.

## References

[ece371490-bib-0001] Abt, K. , and W. Bock . 1998. “Seasonal Variations of Diet Composition in Farmland Field *Apodemus* spp. Mice and Bank Voles *Clethrionomys glareolus* .” Acta Theriologica (Warszawa) 43: 379–389. 10.4098/AT.arch.98-49.

[ece371490-bib-0002] Akinwande, M. O. , H. G. Dikko , and A. Samson . 2015. “Variance Inflation Factor: As a Condition for the Inclusion of Suppressor Variable(s) in Regression Analysis.” Open Journal of Statistics 05: 754–767. 10.4236/ojs.2015.57075.

[ece371490-bib-0003] Amori, G. , V. Castigliani , O. Locasciulli , and L. Luiselli . 2015. “Long‐Term Density Fluctuations and Microhabitat Use of Sympatric *Apodemus flavicollis* and *Myodes glareolus* in Central Italy.” Community Ecology 16: 196–205. 10.1556/168.2015.16.2.7.

[ece371490-bib-0004] Amori, G. , M. Cristaldi , A. Fanfani , L. Solida , and L. Luiselli . 2010. “Ecological Coexistence of Low‐Density Populations of *Apodemus sylvaticus* and *A. flavicollis* (Mammalia: Rodentia).” Rendiconti Lincei 21: 171–182. 10.1007/s12210-010-0076-2.

[ece371490-bib-0005] Andrzejewski, R. , and J. Olszewski . 1963. “Social Behaviour and Interspecific Relations in *Apodemus flavicollis* (Melchior, 1834) and *Clethrionomys glareolus* (Schreber, 1780).” Acta Theriologica (Warszawa) 7: 155–168. 10.4098/AT.arch.63-10.

[ece371490-bib-0006] Aulagnier, S. , P. Haffner , A. J. Mitchell‐Jones , F. Moutou , and J. Zima . 2009. Mammals of Europe, North Africa and the Middle East. A. & C. Black.

[ece371490-bib-0007] Bartoń, K. 2023. “MuMIn: Multi‐Model Inference. R Package Version 1.47.5.” https://CRAN.R‐project.org/package=MuMIn.

[ece371490-bib-0008] Bates, D. , M. Maechler , B. Bolker , and S. Walker . 2015. “Fitting Linear Mixed‐Effects Models Using lme4.” Journal of Statistical Software 67: 1–48. 10.18637/jss.v067.i01.

[ece371490-bib-0009] Begon, M. , C. R. Townsend , and J. L. Harper . 2009. Ecology: From Individuals to Ecosystems. Wiley.

[ece371490-bib-0010] Benedek, A. M. , C. Drăgulescu , and M. Drugă . 2004. “Comparative Study of Some Mountain Habitat Types in Retezatul Mic Mountains (Romania) Based on Cormophytes' Ecology.” Contribuții Botanice 39: 59–67.

[ece371490-bib-0011] Benedek, A. M. , and I. Sîrbu . 2018. “Responses of Small Mammal Communities to Environment and Agriculture in a Rural Mosaic Landscape.” Mammalian Biology 90: 55–65. 10.1016/j.mambio.2018.02.008.

[ece371490-bib-0012] Benedek, A. M. , and I. Sîrbu . 2019. “Dynamics of Small‐Mammal Communities Along an Elevational Gradient.” Canadian Journal of Zoology 97: 312–318. 10.1139/cjz-2018-0201.

[ece371490-bib-0013] Benedek, A. M. , I. Sîrbu , and A. Lazăr . 2021. “Responses of Small Mammals to Habitat Characteristics in Southern Carpathian Forests.” Scientific Reports 11: 12031. 10.1038/s41598-021-91488-6. 34103594 PMC8187625

[ece371490-bib-0014] Beyer, H. L. , D. T. Haydon , J. M. Morales , et al. 2010. “The Interpretation of Habitat Preference Metrics Under Use–Availability Designs.” Philosophical Transactions of the Royal Society of London. Series B, Biological Sciences 365: 2245. 10.1098/rstb.2010.0083. 20566501 PMC2894962

[ece371490-bib-0015] Bhattacharyya, S. , S. Dutta , B. S. Adhikari , and G. S. Rawat . 2015. “Presence of a Small Mammalian Prey Species in Open Habitat is Dependent on Refuge Availability.” Mammal Research 60: 293–300. 10.1007/s13364-015-0234-0.

[ece371490-bib-0016] Biedma, L. , J. Calzada , J. A. Godoy , and J. Román . 2020. “Local Habitat Specialization as an Evolutionary Response to Interspecific Competition Between Two Sympatric Shrews.” Journal of Mammalogy 101: 80–91. 10.1093/jmammal/gyz203.

[ece371490-bib-0017] Bista, D. , P. K. Paudel , S. R. Jnawali , A. P. Sherpa , S. Shrestha , and K. P. Acharya . 2019. “Red Panda Fine‐Scale Habitat Selection Along a Central Himalayan Longitudinal Gradient.” Ecology and Evolution 9: 5260–5269. 10.1002/ece3.5116. 31110677 PMC6509368

[ece371490-bib-0018] Boyce, M. S. , C. J. Johnson , E. H. Merrill , S. E. Nielsen , E. J. Solberg , and B. van Moorter . 2016. “Can Habitat Selection Predict Abundance?” Journal of Animal Ecology 85: 11–20. 10.1111/1365-2656.12359. 25786026

[ece371490-bib-0019] Buesching, C. D. , C. Newman , R. Twell , and D. W. Macdonald . 2008. “Reasons for Arboreality in Wood Mice *Apodemus sylvaticus* and Bank Voles *Myodes glareolus* .” Mammalian Biology 73: 318–324. 10.1016/j.mambio.2007.09.009.

[ece371490-bib-0020] Canova, L. 1993. “Resource Partitioning Between the Bank Vole *Clethrionomys glareolus* and the Wood Mouse *Apodemus sylvaticus* in Woodland Habitats.” Bollettino di Zoologia 60: 193–198. 10.1080/11250009309355809.

[ece371490-bib-0021] Čepelka, L. , J. Šipoš , J. Suchomel , and M. Heroldová . 2020. “Can We Detect Response Differences Among Dominant Rodent Species to Climate and Acorn Crop in a Central European Forest Environment?” European Journal of Forest Research 139: 539–548. 10.1007/s10342-020-01267-7.

[ece371490-bib-0022] Connell, J. H. 1980. “Diversity and the Coevolution of Competitors, or the Ghost of Competition Past.” Oikos 35: 131–138. 10.2307/3544421.

[ece371490-bib-0023] Dormann, C. F. , J. Elith , S. Bacher , et al. 2013. “Collinearity: A Review of Methods to Deal With It and a Simulation Study Evaluating Their Performance.” Ecography 36: 27–46. 10.1111/j.1600-0587.2012.07348.x.

[ece371490-bib-0024] Dueser, R. D. , and J. G. Hallett . 1980. “Competition and Habitat Selection in a Forest‐Floor Small Mammal Fauna.” Oikos 35: 293–297. 10.2307/3544642.

[ece371490-bib-0025] Flowerdew, J. R. , T. Amano , and W. J. Sutherland . 2017. “Strong “Bottom‐Up” Influences on Small Mammal Populations: State‐Space Model Analyses From Long‐Term Studies.” Ecology and Evolution 7: 1699–1711. 10.1002/ece3.2725. 28331581 PMC5355190

[ece371490-bib-0026] Fortin, D. , R. Courtois , P. Etcheverry , C. Dussault , and A. Gingras . 2008. “Winter Selection of Landscapes by Woodland Caribou: Behavioural Response to Geographical Gradients in Habitat Attributes.” Journal of Applied Ecology 45: 1392–1400. 10.1111/j.1365-2664.2008.01542.x.

[ece371490-bib-0027] Fretwell, S. D. 1972. Populations in a Seasonal Environment. Princeton University Press. 4680650

[ece371490-bib-0028] Gillingham, M. P. , and K. L. Parker . 2008. “Differential Habitat Selection by Moose and Elk in the Besaprophet Area of Northern British Columbia.” Alces: A Journal Devoted to the Biology and Management of Moose 44: 41–63.

[ece371490-bib-0029] Greenwood, P. J. 1978. “Timing of Activity of the Bank Vole *Clethrionomys glareolus* and the Wood Mouse *Apodemus sylvaticus* in a Deciduous Woodland.” Oikos 31: 123–127. 10.2307/3543393.

[ece371490-bib-0030] Harrell, F. E. 2023. “Hmisc: Harrell Miscellaneous. R Package Version 5.1–1.” https://CRAN.R‐project.org/package=Hmisc.

[ece371490-bib-0031] Hille, S. M. , and A. Mortelliti . 2011. “Microhabitat Partitioning of *Apodemus flavicollis* and *Myodes glareolus* in the Sub‐Montane Alps: A Preliminary Assessment.” Hystrix, the Italian Journal of Mammalogy 21: 157–163. 10.4404/hystrix-21.2-4458.

[ece371490-bib-0032] Johnson, D. H. 1980. “The Comparison of Usage and Availability Measurements for Evaluating Resource Preference.” Ecology 61: 65–71. 10.2307/1937156.

[ece371490-bib-0033] Jorgensen, E. E. 2004. “Small Mammal Use of Microhabitat Reviewed.” Journal of Mammalogy 85: 531–539. 10.1644/BER-019.

[ece371490-bib-0034] Kawaguchi, T. , and A. Desrochers . 2018. “A Time‐Lagged Effect of Conspecific Density on Habitat Selection by Snowshoe Hare.” PLoS One 13: e0190643. 10.1371/journal.pone.0190643. 29320564 PMC5761860

[ece371490-bib-0035] Kelt, D. A. , E. J. Heske , X. Lambin , et al. 2019. “Advances in Population Ecology and Species Interactions in Mammals.” Journal of Mammalogy 100: 965–1007. 10.1093/jmammal/gyz017.

[ece371490-bib-0036] Kramer, H. A. , G. M. Jones , V. R. Kane , et al. 2021. “Elevational Gradients Strongly Mediate Habitat Selection Patterns in a Nocturnal Predator.” Ecosphere 12: e03500. 10.1002/ecs2.3500.

[ece371490-bib-0037] Lehtonen, J. T. , O. Mustonen , H. Ramiarinjanahary , J. Niemelä , and H. Rita . 2001. “Habitat Use by Endemic and Introduced Rodents Along a Gradient of Forest Disturbance in Madagascar.” Biodiversity and Conservation 10: 1185–1202. 10.1023/A:1016687608020.

[ece371490-bib-0038] Lüdecke, D. , M. S. Ben‐Shachar , I. Patil , P. Waggoner , and D. Makowski . 2021. “Performance: An R Package for Assessment, Comparison and Testing of Statistical Models.” Journal of Open Source Software 6, no. 60: 3139. 10.21105/joss.03139.

[ece371490-bib-0039] Lyu, S. , and J. M. Alexander . 2022. “Competition Contributes to Both Warm and Cool Range Edges.” Nature Communications 13: 2502. 10.1038/s41467-022-30013-3. PMC9076896 35523780

[ece371490-bib-0040] Mandeville, C. P. , A. G. Finstad , J. A. Kålås , B. G. Stokke , I. J. Øien , and E. B. Nilsen . 2024. “Interspecific Competition Impacts the Occupancy and Range Limits of Two Ptarmigan Species Along the Elevation Gradient in Norway.” Wildlife Biology 2024: e01197. 10.1002/wlb3.01197.

[ece371490-bib-0041] Marsh, A. C. W. , S. Poulton , and S. Harris . 2001. “The Yellow‐Necked Mouse *Apodemus flavicollis* in Britain: Status and Analysis of Factors Affecting Distribution.” Mammal Review 31: 203–227. 10.1111/j.1365-2907.2001.00089.x.

[ece371490-bib-0042] Mayor, S. , D. Schneider , J. Schaefer , and S. Mahoney . 2009. “Habitat Selection at Multiple Scales.” Ecoscience 16: 238–247. 10.2980/16-2-3238.

[ece371490-bib-0043] Montgomery, W. I. 1980. “The Use of Arboreal Runways by the Woodland Rodents, *Apodemus sylvaticus* (L.), *A. flavicollis* (Melchior) and *Clethrionomys glareolus* (Schreber).” Mammal Review 10: 189–195. 10.1111/j.1365-2907.1980.tb00239.x.

[ece371490-bib-0044] Morris, D. W. , and A. Dupuch . 2012. “Habitat Change and the Scale of Habitat Selection: Shifting Gradients Used by Coexisting Arctic Rodents.” Oikos 121: 975–984. 10.1111/j.1600-0706.2011.20492.x.

[ece371490-bib-0045] Morris, D. W. , and J. T. MacEachern . 2010. “Active Density‐Dependent Habitat Selection in a Controlled Population of Small Mammals.” Ecology 91: 3131–3137. 10.1890/10-0479.1. 21141174

[ece371490-bib-0046] Naxara, L. , B. T. Pinotti , and R. Pardini . 2009. “Seasonal Microhabitat Selection by Terrestrial Rodents in an Old‐Growth Atlantic Forest.” Journal of Mammalogy 90: 404–415. 10.1644/08-MAMM-A-100.1.

[ece371490-bib-0047] Pimm, S. L. 1985. “Estimating Competition Coefficients From Census Data.” Oecologia 67: 588–590. 28311045 10.1007/BF00790031

[ece371490-bib-0064] R Core Development Team . 2023. R: A language and environment for statistical computing, version 4.3.0. R Foundation for Statistical Computing. http://www.r‐project.org.

[ece371490-bib-0048] Rosenzweig, M. L. 1981. “A Theory of Habitat Selection.” Ecology 62: 327–335. 10.2307/1936707.

[ece371490-bib-0049] Rosenzweig, M. L. , and Z. Abramsky . 1985. “Detecting Density‐Dependent Habitat Selection.” American Naturalist 126: 405–417.

[ece371490-bib-0050] Selva, N. , K. A. Hobson , A. Cortés‐Avizanda , A. Zalewski , and J. A. Donázar . 2012. “Mast Pulses Shape Trophic Interactions Between Fluctuating Rodent Populations in a Primeval Forest.” PLoS One 7: e51267. 10.1371/journal.pone.0051267. 23251475 PMC3519590

[ece371490-bib-0051] Shenbrot, G. 2004. “Habitat Selection in a Seasonally Variable Environment: Test of the Isodar Theory With the Fat Sand Rat, *Psammomys obesus* , in the Negev Desert, Israel.” Oikos 106: 359–365. 10.1111/j.0030-1299.2004.13123.x.

[ece371490-bib-0052] Sirén, A. P. K. , and T. L. Morelli . 2020. “Interactive Range‐Limit Theory (iRLT): An Extension for Predicting Range Shifts.” Journal of Animal Ecology 89: 940–954. 10.1111/1365-2656.13150. 31758805 PMC7187220

[ece371490-bib-0053] Sozio, G. , and A. Mortelliti . 2016. “Empirical Evaluation of the Strength of Interspecific Competition in Shaping Small Mammal Communities in Fragmented Landscapes.” Landscape Ecology 31: 775–789. 10.1007/s10980-015-0286-1.

[ece371490-bib-0054] Stoffel, M. A. , S. Nakagawa , and H. Schielzeth . 2021. “partR2: Partitioning R2 in Generalized Linear Mixed Models.” PeerJ 9: e11414. 10.7717/peerj.11414. 34113487 PMC8162244

[ece371490-bib-0055] Suárez, O. V. , and S. M. Bonaventura . 2001. “Habitat Use and Diet in Sympatric Species of Rodents of the Low Paraná Delta, Argentina.” Mammalia 65: 167–176. 10.1515/mamm.2001.65.2.167.

[ece371490-bib-0056] Suárez‐Gracida, C. G. , and S. T. Álvarez‐Castañeda . 2009. “Physical and Biological Variables Related to Habitat Preferences of Rodents.” Biodiversity and Conservation 18: 2779–2797. 10.1007/s10531-009-9606-6.

[ece371490-bib-0057] Sundell, J. , C. Church , and O. Ovaskainen . 2012. “Spatio‐Temporal Patterns of Habitat Use in Voles and Shrews Modified by Density, Season and Predators.” Journal of Animal Ecology 81: 747–755. 10.1111/j.1365-2656.2012.01956.x. 22325037

[ece371490-bib-0058] Torre, I. , and A. Arrizabalaga . 2008. “Habitat Preferences of the Bank Vole *Myodes glareolus* in a Mediterranean Mountain Range.” Acta Theriologica 53: 241–250. 10.1007/BF03193120.

[ece371490-bib-0059] Väli, Ü. , and G. Tõnisalu . 2020. “Community‐ and Species‐Level Habitat Associations of Small Mammals in a Hemiboreal Forest–Farmland Landscape.” Annales Zoologici Fennici 58: 1–11. 10.5735/086.058.0101.

[ece371490-bib-0060] Villanueva‐Hernández, A. I. , D. A. Delgado‐Zamora , S. A. Heynes‐Silerio , L. Ruacho‐González , and C. López‐González . 2017. “Habitat Selection by Rodents at the Transition Between the Sierra Madre Occidental and the Mexican Plateau, México.” Journal of Mammalogy 98: 293–301. 10.1093/jmammal/gyw173.

[ece371490-bib-0061] Viviano, A. , M. Scarfò , and E. Mori . 2022. “Temporal Partitioning Between Forest‐Dwelling Small Rodents in a Mediterranean Deciduous Woodland.” Animals 12: 279. 10.3390/ani12030279. 35158603 PMC8833473

[ece371490-bib-0062] Wójcik, J. M. , and K. Wołk . 1985. “The Daily Activity Rhythm of Two Competitive Rodents: *Clethrionomys glareolus* and *Apodemus flavicollis* .” Acta Theriologica (Warszawa) 30: 241–258. 10.4098/AT.arch.85-16.

[ece371490-bib-0063] Zwolak, R. , J. Witczuk , M. Bogdziewicz , L. Rychlik , and S. Pagacz . 2018. “Simultaneous Population Fluctuations of Rodents in Montane Forests and Alpine Meadows Suggest Indirect Effects of Tree Masting.” Journal of Mammalogy 99: 586–595. 10.1093/jmammal/gyy034.

